# The role of short-chain fatty acids in the interplay between gut microbiota and diet in cardio-metabolic health

**DOI:** 10.1080/19490976.2021.1897212

**Published:** 2021-03-25

**Authors:** Ana Nogal, Ana M. Valdes, Cristina Menni

**Affiliations:** a Department of Twin Research, King’s College London, St Thomas’ Hospital Campus, London, UK; b School of Medicine, Nottingham City Hospital, Nottingham, UK; c NIHR Nottingham Biomedical Research Centre, Nottingham, UK

**Keywords:** Short-chain fatty acids, gut microbiota, diet, cardio-metabolic health, omega-3, fiber

## Abstract

The gut microbiota plays an important role in cardio-metabolic diseases with diet being among the strongest modulators of gut microbiota composition and function. Resistant dietary carbohydrates are fermented to short-chain fatty acids (SCFAs) by the gut bacteria. Fiber and omega-3 rich diets increase SCFAs production and abundance of SCFA-producing bacteria. Likewise, SCFAs can improve gut barrier integrity, glucose, and lipid metabolism, regulate the immune system, the inflammatory response, and blood pressure. Therefore, targeting the gut microbiota with dietary strategies leading to increased SCFA production may benefit cardio-metabolic health. In this review, we provide an overview of the association between diet, SCFAs produced by the gut microbiota and cardio-metabolic diseases. We first discuss the association between the human gut microbiota and cardio-metabolic diseases, then investigate the role of SCFAs and finally explore the beneficial effects of specific dietary interventions that can improve cardio-metabolic outcomes through boosting the SCFA production.

## Introduction

Cardio-metabolic diseases (CMD) are the most common cause of morbidity and mortality worldwide, representing a major public health challenge.^1^^; 2^ Indeed, 25% of the total population is estimated to have CMD, and approximately 30% of all the deaths are caused by CMDs.^3^

There are many well-established genetic and environmental risk factors associated to CMD including high blood pressure, high cholesterol, smoking, diabetes, abdominal obesity, insulin resistance, glucose intolerance among others. Recent studies suggest that gut microbiota imbalance also plays an important role in the development and progression of CMDs,^4–7^ including type-2 diabetes (T2D), obesity, atherosclerosis, heart failure, myocardial fibrosis, and atrial fibrillation. Moreover, the gut-brain axis has been implicated in neurogenic hypertension,^8^ which is particularly relevant for people suffering from obstructive sleep apnea,^9^ and gut microbiome composition has been linked to sleep architecture in these patients.^10^ Given the strong link between inflammation and sleep apnea, it is likely that SCFAs/gut microbiome links relating to hypertension may also link to sleep apnea and other disorders.^11^

Gut microbiota can exert beneficial or detrimental effects in human health^12^ through the production of metabolic products and signaling molecules, which influence diverse functions in different organs.^13^ Among these bacteria-derived metabolites, short-chain fatty acids (SCFA) are gaining attention as a potential focus of CMD.^14^ SCFAs, namely acetate, propionate, and butyrate, are produced through bacterial fermentation of fibers (e.g., resistant starch, simple sugars, and polysaccharides),^15^ and present regulatory functions in the lipids, cholesterol and glucose metabolism, anti-inflammatory and immune response and gut barrier integrity.^16^ SCFAs might protect against CMD as they are able, for instance, to decrease plasma cholesterol and glucose levels and increase fatty acid oxidation.^17^ Likewise, the type of diet can modulate SCFA production and/or abundance of SCFA-producing bacteria. For instance, a Mediterranean diet, characterized by a high consumption of fiber-rich food, or omega-3-rich diets, has been correlated with higher levels of SCFAs and SCFA-producing bacteria.^18,19^ Previous reviews addressed SCFAs, CMD or, diet and their links to gut microbiome composition,^20–25^ however none of them has yet simultaneously covered the role of SCFAs in the interplay between diet, gut microbes, and CMD. Indeed, most of the previous reviews do not discuss in detail SCFAs^23^ nor their beneficial role in cardio-metabolic health,^20^ nor how different types of diet can increase SCFAs production to improve the health of patients suffering from CMDs.^21,22,24,25^ In the present review, on the contrary, we specifically focus on the association between the human gut microbiota and CMDs, the metabolic routes involved in the SCFA production, the benefits exerted by SCFAs in cardio-metabolic health, and the involved mechanisms. Finally, we discuss a number of recent clinical studies supporting the beneficial effects of specific dietary interventions that can improve cardio-metabolic outcomes through boosting SCFAs production.

## Gut microbiota

The human intestine contains approximately 10^14^ microorganisms, collectively known as the gut microbiota.^26^ The gene repertoire present in these microbes is 100-fold higher than the number of genes present in the human genome.^27^ In a healthy gut microbiota, the most predominant phyla are Firmicutes and Bacteroidetes (90% of the population), followed by Actinobacteria and Verrucomicrobia,^28^ although inter-variability between individuals exists. Gut microbiota diversity, richness, and composition vary depending on multiple determinants, either endogenous such as sex, microbial interactions, and host genotype,^26,29^ or exogenous, such as diet, age, usage of antibiotics, exercise, smoking, and stress.^30^

Over the last years, advances in bioinformatics tools and next-generation sequencing have increased our knowledge on the relationship between microbiota organisms and humans,^31^ allowing us to discover the benefits and detriments of the gut bacteria to human health. Bacteria-derived metabolites play important functions in the intestine (e.g., digestion, energy harvest, and barrier integrity)^32^ and even in other organs when they enter into the systemic circulation (e.g., glucose circulation in the pancreas, lipid metabolism in the liver, and cognitive functions in the brain).^13^ When there is an intestinal microbial ecosystem balance (eubiosis), the gut microbiota plays important immunological, homeostatic, and metabolic functions that maintain the human host health.^33^ On the other hand, the imbalance of the gut microbiota, known as dysbiosis, and reduction of bacterial diversity can lead to a variety of metabolic abnormalities, such as inflammation and oxidative stress, impacting negatively on the host pathophysiologic and physiology conditions.^34^

## Gut microbiota in cardio-metabolic health

The role of the gut microbiota has recently been implicated in the development and progression of CMD.^35,36^ Many studies have shown alterations in the composition and function of the gut bacteria in patients suffering from CMDs. Animal and human obesity has been associated with an increased Firmicutes/Bacteroidetes ratio.^37^ Moreover, gut dysbiosis can reduce the gut barrier integrity, affecting glucose sensibility and absorption, leading to insulin resistance and T2D.^38^ Another example is the lipopolysaccharides (LPS) present in the Gram-negative bacteria cell wall, which can trigger the immune system response and potentiate cardiovascular diseases (CVD) pathogenesis.^39,40^

Our body can functionally interact with bacteria metabolic products,^16^ and cardio-metabolic health (CMH) is associated with these metabolic products. Trimethylamine (TMA),^41–45^ bile acids^46–49^ are examples of metabolic products that have been negatively associated with CMD, whereas SCFAs,^14^ anthocyanins^50^ and indoleproprionic acid^51^ might influence positively the host health.

TMA is metabolized from choline-containing compounds (e.g., choline, betaine, and L-carnitine) present in human diet by the gut microbiota.^52^ Then, TMA enters to the portal circulation, where is oxidized by liver enzymes to produce trimethylamine‑N‑oxide (TMAO).^53^ TMAO pathway has been associated with atherosclerosis and thrombosis promotion in mouse,^41–43^ and with CMD in humans such as obesity, chronic kidney disease, and T2D.^44,45,54^ Detailed therapeutic potential and clinical prognostic of TMAO in CMD can be found in several reviews.^55–57^

Gut microbiota is responsible for the generation of unconjugated free bile acids and secondary bile acids through deconjugation and dihydroxylation reactions.^58^ Bile acids can act as signaling molecules involved in inflammation, host metabolism, and energy expenditure, and thus, they might play a role in CMD.^46–49^ The role of bile acids in metabolic disorders and CVD has been reviewed by.^59,60^

Anthocyanins are glycosyl-anthocyanidins present in plant vacuoles. Gut bacteria can degrade anthocyanins, generating protocatechuic acid and free anthocyanidins.^61^ Protocatechuic acid can influence positively atherosclerosis and CVD, thanks to its anti-inflammatory and antioxidant properties.^50^

Indoleproprionic acid is a compound synthetized from tryptophan by a reduced number of bacterial strains.^62^ Circulating levels of indoleproprionic acid are negatively correlated with different metabolic syndrome parameters,^51^ and higher levels of this compound have been also associated with a lower risk of developing T2D.^63^

SCFAs are the most well-studied gut bacteria-derived metabolites and they have been suggested as potential disease-mitigating factors and/or disease preventing in CMD, including T2D, obesity, and CVD, among others.^14,64^ SCFAs will be explained in detail in the below sections.

Hence, CMD development might be modulated via specific beneficial bacteria-derived metabolites. Likewise, dietary interventions can have a profound effect on their production,^65,66^ being very important to investigate the role of these metabolites on human health and their modulation by different diets.

## Short-chain fatty acids

Fatty acids are carboxylic acids with an aliphatic chain, which can be saturated or unsaturated.^67^ Depending on the length of their aliphatic tails, fatty acids can be classified as short (<6 C), medium (6–12 C), or long (>12 C) chain fatty acids. SCFAs include formate (C1), acetate (C2), propionate (C3), butyrate (C4), and valerate (C5), and their chemical properties depend on the number of carbons.^68^

SCFAs are produced by anaerobic gut bacteria through saccharolytic fermentation of complex resistant carbohydrates (e.g., fructo-oligosaccharides, sugar alcohols, resistant starch, inulin, and polysaccharides from plant cell walls), which escape digestion and absorption in the small intestine.^69^ As a result of the fermentative reactions, some gases, including hydrogen, methane, and carbon dioxide are generated.^70^ It is estimated that the fermentation of 50–60 g carbohydrates per day yields to the approximated production of 500–600 mmol of SCFAs in the gut.^71^ Amino acids can be also fermented to produce SCFAs.^72^ Although SCFAs are dependent on diet and bacteria present in the gut, there are specific foods containing SCFAs, for instance, vinegar, sourdough bread, and some dairy products such as crème fraiche, butter, and cheese.^73^

**Figure 1.**
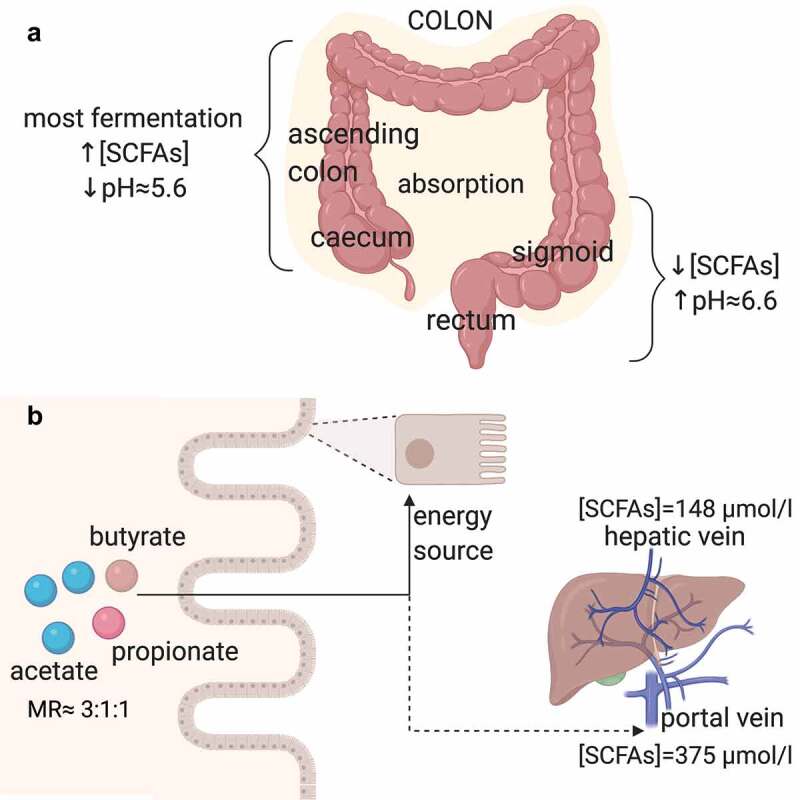
Overview of the production and absorption sites, and transport of acetate, propionate and butyrate (SCFAs)

The major SCFAs formed by the gut bacteria are acetate, propionate, and butyrate which account for approximately 80% of all SCFAs and will be the focus of this review. In order to comprehensively understand the effect of these metabolites on human health, it is essential to consider the production site and the gradient along different cells and tissues (**Figure 1**). Fermentation takes place in the large intestine, mainly at the right side, and the SCFA absorption occurs rapidly from the human colon.^74^ Changes in pH vary depending on the SCFA concentration.^75^ In the cecum, the pH is more acidic and the SCFA concentrations are higher than in the sigmoid/rectum, where the pH is higher. In the colon and stool, butyrate, propionate, and acetate are found in an approximate molar ratio of 20:20:60, respectively,^76^ although these values vary depending on the microbiota composition, SCFA substrates, and gut transit time.^77^ Additionally, a strong gradient from the gut lumen to the periphery exists, leading to different cell SCFA exposure.^78^ Most SCFAs are utilized by colonocytes as an energy source.^71^ The SCFAs that are not used by these cells can be transported toward the hepatic portal vein. Acetate, propionate, and butyrate concentrations in portal blood (375 µmol/l) are almost 5 times greater than peripheral venous blood (79 µmol/l), suggesting that the gut is a principal SCFA source, whereas SCFA concentrations in the hepatic vein SCFA (148 µmol/l) are 39% of those found in portal blood.^76^

(A) Most undigested carbohydrates are fermented in the cecum and ascending colon, whereas the SCFA absorption takes place along the whole colon. A negative correlation between the SCFA concentrations and pH exists. The highest SCFA concentration levels are in the cecum and ascending colon, where the pH is approximately 5.6, whereas in the sigmoid and rectum, the pH is higher (approximately 6.6) and the SCFA concentrations are lower. (B) In the colon, acetate, propionate, and butyrate are found in an approximate molar ratio of 3:1:1, respectively. Most SCFAs are utilized by colonocytes as an energy source. The SCFAs that are not used by these cells can be transported toward the hepatic portal vein, where the SCFA concentrations are 375 µmol/l, and the hepatic vein, where the SCFA concentrations are 39% of those found in portal blood. MR, molar ratio. SCFAs, short-chain fatty acids. Created with BioRender.com

## Metabolic routes to produce SCFA and SCFA-producing bacteria

**Figure 2.**
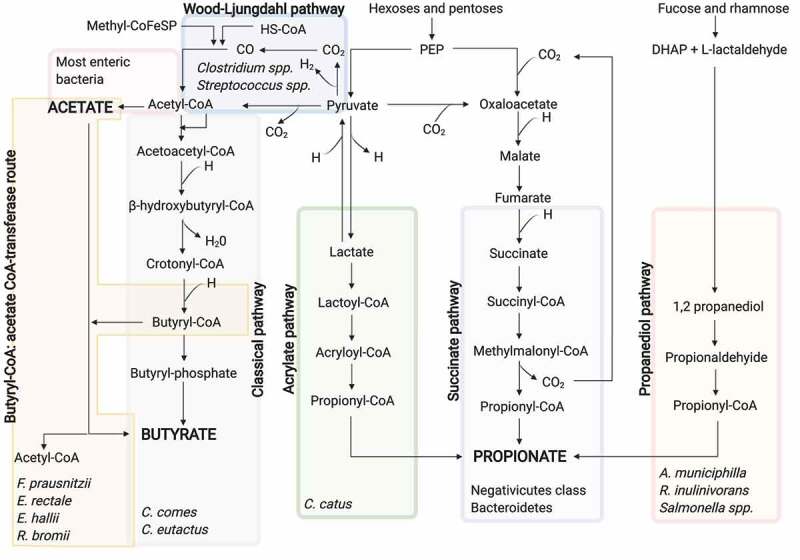
SCFA biosynthesis pathways from the dietary carbohydrate fermentation and the major SCFA-producing bacteria for each pathway

The pathways involved in the SCFA production have been recently described in detail.^79^ In addition, metagenomic analyses have allowed the characterization of the major SCFA-producing bacteria^21^ (**Figure 2**).

Acetate can be formed by the Wood-Ljungdahl pathway and from pyruvate via acetyl-CoA. Acetyl-CoA can be also produced from lactate by lactate-utilizing bacteria. Three pathways exist for the propionate formation, namely acrylate, succinate, and propanediol pathways. The two first use PEP and the latter utilizes deoxy sugars such as rhamnose and fucose. Butyrate can be formed through the classical pathway from the condensation of two acetyl-CoA molecules or by the butyryl-CoA: acetate CoA-transferase route, in which butyryl-CoA is converted into butyrate and acetyl-CoA using exogenously derived acetate. DHAP, dihydroxyacetone phosphate; PEP, phosphoenolpyruvate. Created with BioRender.com

### Acetate formation

Acetate can be synthesized through two different pathways. Firstly, acetyl-CoA can be produced by decarboxylation of pyruvate, then, acetyl-CoA is hydrolyzed to acetate by an acetyl-CoA hydrolase.^80^ Most of the acetate is produced by enteric bacteria, including *Prevotella* spp., *Ruminococcus* spp., *Bifidobacterium* spp., *Bacteroides* spp., *Clostridium* spp., *Streptococcus* spp., *A. muciniphila*, and *B. hydrogenotrophica*, using this pathway.^81^ Secondly, the *Wood-Ljungdahl* pathway can be also used by acetogenic bacteria to form acetate from acetyl-CoA. Here, the reduction of carbon dioxide generates carbon monoxide, which reacts with a coenzyme A molecule and a methyl group to produce acetyl-CoA. At the same time, acetyl-CoA is the substrate to obtain acetate.^82^

### Propionate formation

Although propionate-producers are distributed across several phyla, only a few bacterial genera are able to form propionate, and unlike acetate, the utilized propionate pathways are more conserved and substrate specific.^83^

Propionate can be synthesized through three different biochemical pathways, namely succinate, acrylate, and propanediol pathway.^83^ In the succinate pathway, the primitive electron transfer chain using phosphoenolpyruvate (PEP) can be utilized to generate propionate.^84^ Specifically, PEP is carboxylated to oxalacetate, and then oxalacetate is sequentially converted into malate and fumarate. The latter accepts electrons from NADH using a fumarate reductase and a NADH dehydrogenase, which form a simple electron-transfer chain. The NADH dehydrogenase transport protons across the cell membrane. These protons are utilized for chemiosmotic ATP synthesis. Likewise, succinate is generated as a result of the fumarate reductase. When the carbon dioxide partial pressure is low, succinate is transformed to methylmalonate, which leads to propionate and carbon dioxide. The latter can be recycled for the PEP carboxylation, repeating the process. Bacteroidetes^85^ and several Firmicutes belonging to the Negativicutes class^86^ use this pathway for the propionate formation. Besides, acrylate pathway can be used to reduce lactate to propionate by a lactoyl-CoA dehydratase.^80^ This pathway is only present in a very reduced number of gut bacteria, including *Coprococcus catus*.^83^ Lastly, 1,2-propanediol can be formed from deoxy sugars such as rhamnose and fucose in the propanediol pathway. Likewise, 1,2-propanediol is sequentially converted into propionaldehyde and propionyl-CoA, which leads to the propionate formation.^87^ *Salmonella enterica* serovar Typhimurium^88^ and *R. inulinivorans*^89^ are bacteria utilizing this pathway, just as *Akkermansia municiphilla* which appears to be the major propionate-producing species.^90^

### Butyrate formation

Butyrate production, like propionate, is more conserved and substrate specific.^83^ Resistant starch fermentation highly contributes to the formation of butyrate in the colon, with *Ruminococcus bromii* the main producer as its absence has been associated with a reduction in the resistant starch fermentation.^91^

To form butyrate, first, two acetyl-CoA molecules must be condensed to obtain acetoacetyl-CoA, which is subsequently reduced to β-hydroxybutyryl-CoA, crotonyl-CoA and lastly to butyryl-CoA. In the case of lactate-utilizing bacteria, acetyl-CoA can be produced from lactate.^92^ From butyryl-CoA, butyrate can be synthesized following two different pathways. In the pathway referred to as classical, phosphotransbutyrylase and butyrate kinase enzymes are responsible for such a conversion.^93^ In the second pathway, butyryl-CoA: acetate CoA-transferase converts butyryl-CoA into butyrate and acetyl-CoA using exogenously derived acetate. The latter pathway seems to be preferred by the human gut microbiota rather than the classical pathway,^94^ which is limited to some *Coprococcus* species.^79^ *F. prausnitzii, E. rectale, E. hallii*, and *R. bromii* present this pathway and appear to be the major butyrate producers.^95^

## Beneficial roles of SCFA in cardio-metabolic health and involved mechanisms

**Figure 3.**
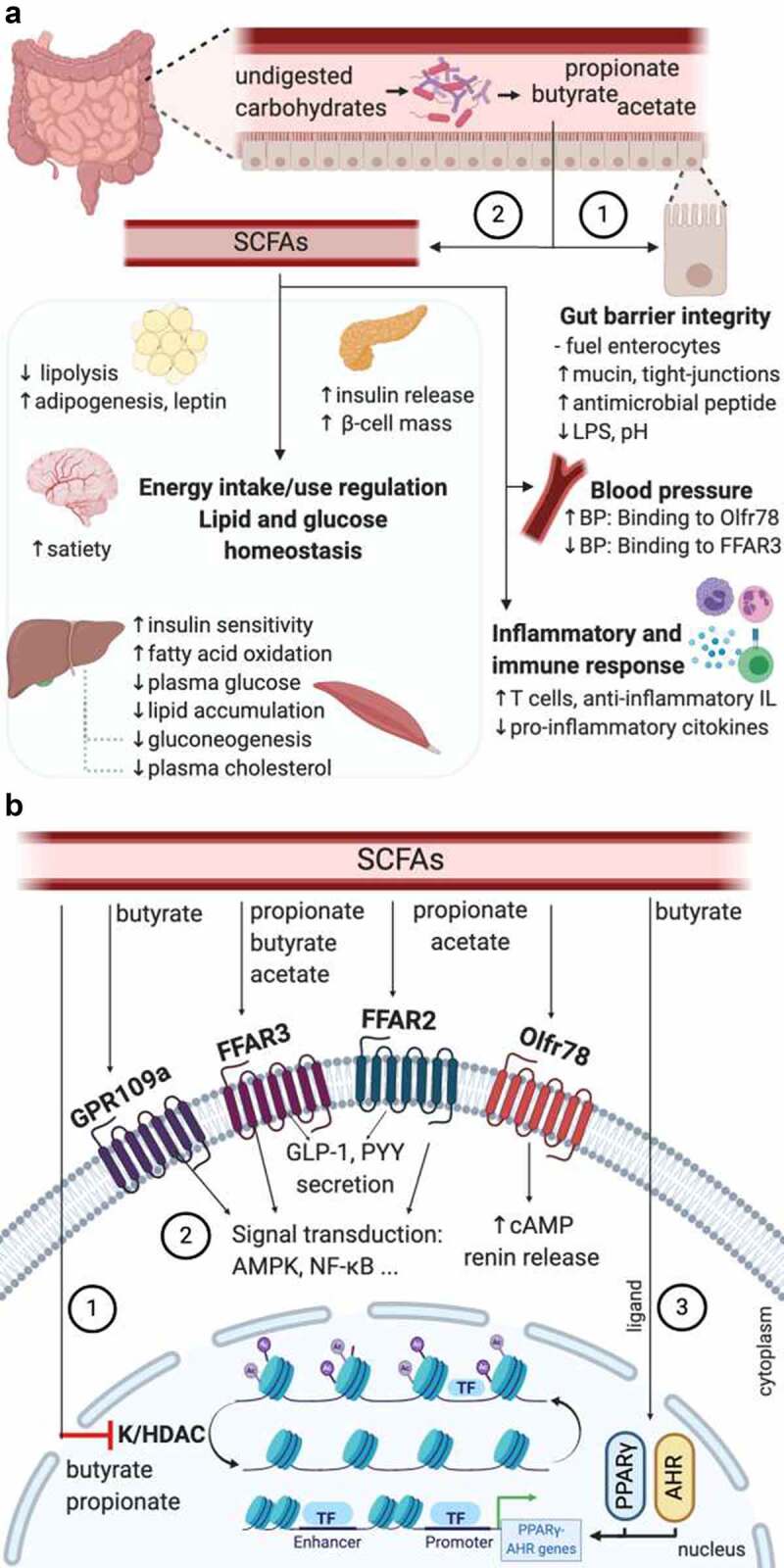
Beneficial roles of SCFA in cardio-metabolic health and the indirect mechanisms involved

SCFAs act as signaling molecules on both the gut cells and other tissue cells. This is possible due to six receptors to which SCFAs can bind, triggering intracellular signaling cascades: free fatty acid receptor 3 (FFAR3 or GPR41), FFAR2 (also known as GRP43), G-protein coupled receptor 109a (GPR109a or HCAR2), olfactory receptor-78 (Olfr78 in mice or OR51E2 in humans), GPR42 and OR51E1, being the four first the most well-studied.^16^ Olfr78 mainly binds acetate and propionate, leading to an increase of cyclic adenosine monophosphate (cAMP) and renin release^96^ and is expressed in the vascular smooth muscle cells in the peripheral vasculature and renal afferent arteriole.^97^ FFAR3, FFAR2, and GPR109a are expressed by different organs and cells: small intestine, colon, liver, spleen, heart, skeletal muscle, neurons, immune cells, and adipose tissues.^98^ Additionally, depending on the length of their aliphatic tails, the receptors present different affinities for SCFAs. FFAR2 prefers binding to acetate and propionate, whereas FFAR3 binds propionate, butyrate, and acetate with a lower affinity,^99^ and GPR109a mainly binds butyrate.^100^ Moreover, butyrate and propionate play an important role in transcriptional regulations and post-translational modifications, as they appear to strongly inhibit lysine and histone deacetylase (K/HDAC) activity.^16,101^ Such an inhibition leads to histone hyperacetylation, which turns in a higher accessibility of transcription factors to the promoter regions of different genes.^102^ Likewise, butyrate is a ligand of two transcription factors: peroxisome proliferator-activated receptor γ (PPARγ)^103^ and aryl hydrocarbon receptor.^104^ Thanks to these and direct mechanisms, SCFAs can play beneficial roles in human health, such as improvement of gut barrier integrity, regulation of the blood pressure and energy intake and energy use, modulation of glucose and lipid metabolism, and mediation of the immune system and anti-inflammatory response (**Figure 3**).

(A) Undigested carbohydrates reach the intestine, where they are fermented by the SCFA-producing bacteria generating acetate, propionate, and butyrate. SCFAs can act using two different mechanisms: 1) direct action on the enterocytes, maintaining the gut barrier integrity or 2) indirect action regulating the inflammatory and immune response, blood pressure, energy intake and use, and lipid and glucose homeostasis, through the mechanisms illustrated in (B). (B) 1) Inhibition of K/HDAC leads to histone hyperacetylation, which turns in a higher accessibility of transcription factors to the promoter regions of different genes; 2) signaling transduction activation (in the small intestine, colon, liver, spleen, heart, skeletal muscle, neurons, immune cells, and adipose tissues), and GLP-1 and PYY secretion (in intestinal enteroendocrine L-cells) caused by the binding of SCFAs to the G protein-coupled receptors, and increase of cAMP levels by the binding of propionate or acetate to the receptor Olfr78/OR51E2 (in vascular smooth muscle cells in the peripheral vasculature and renal afferent arteriole). GLP-1 and PYY enter into the systematic circulation exerting benefits in different tissues and cells; 3) butyrate working as a ligand of the AHR and PPARγ, leading to the expression of genes dependent on these two transcription factors. AMPK, AMP-activated protein kinase; AHR, aryl hydrocarbon receptor; BP, blood pressure; cAMP, cyclic adenosine monophosphate; FFAR, free fatty acid receptor; GLP-1, glucagon-like peptide-1; GPR109a, G-protein coupled receptor-109a; IL, interleukins; K/HDAC, lysine/histone deacetylase; LPS, lipopolysaccharides; NF-κB, nuclear factor kappa β; Olfr78, olfactory receptor-78; PYY: peptide YY; SCFA, short-chain fatty acid; TF, transcription factor. Created with BioRender.com

### Blood pressure

Several recent studies have analyzed the association between SCFAs and blood pressure. The results seem contradictory, as some studies have reported that SCFAs can cause hypertension,^105,106^ and others have shown that SCFAs can decrease blood pressure.^107,108^ For instance, studies with Olfr78 null mice present lower blood pressure compared with the wild-type mice.^106^ On the other hand, FFAR3 KO mice are hypertensive.^107^ The potential dual effect of both receptors in the regulation of blood pressure has been analyzed by Pluznick et al. (2013).^97^ In this study, blood pressure was measure in Olfr78 null mice and wild-type mice before and after an antibiotic treatment. After the treatment, the SCFA levels decreased. Moreover, the wild-type mice presented a slight increase in blood pressure due to the decreased SCFA levels, whereas the Olfr78 null mice had a higher blood pressure increase. Thus, the signaling of both receptors in the wild-type mice can better balance the blood response even with lower SCFA levels, whereas Olfr78 null mice presents only the FFAR3 signaling, which results in a higher blood pressure response. Besides, it is important to note that the ligand affinity of Olfr78 for SCFAs is lower than the one presented in FFAR3.^109^ Taken together, the SCFA receptors Olfr78 and FFAR3 might play an opposing role in the regulation of blood pressure, balancing each other to have a healthy blood pressure.^97^ Moreover, using a Mendelian Randomization approach, Sun and coworkers^110^ reported T2D may causally affect hypertension and particularly higher systolic blood pressure. On the other hand, the relationship from hypertension to T2D is unlikely to be causal, highlighting the importance of keeping an optimal glycemic profile in general populations. Therefore, SCFAs might also be beneficial in hypertension by improving glucose homeostasis.^111,112^

### Gut barrier integrity

It is well recognized that SCFAs are necessary substrates for the colonic epithelium maintenance, with butyrate being the preferred oxidative fuel by colonocytes.^113^ Butyrate can induce proliferation in normal colonocytes, but also terminal differentiation and apoptosis in neoplastic cells. This dual role is known as the “butyrate paradox” or “Warburg effect”.^101,114^ Additionally, intestinal epithelial cells (IEC) are connected by transmembrane proteins, namely, tight-junctions, adherent junctions, and desmosomes. SCFAs, in particular butyrate, seem to improve the epithelial barrier integrity by regulating the tight-junction integrity. Several in vitro and experimental animal studies have examined the impact of SCFAs on tight junctions. A study using differentiated IEC observed that butyrate improved the gut barrier integrity through the expression increase of the tight-junctions claudin-1.^115^ An increased expression of other tight-junctions, including claudin-7, ZO-1, ZO-2, occluding and junctional adhesion molecule A (JAMA), was associated with SCFA production in a mice model study.^116^ Butyrate also influences the epithelial O_2_ consumption, contributing to the stabilization of transcription factor hypoxia-inducible factor (HIF), which coordinates the gut barrier protection.^117^ A proper gut barrier integrity is essential to avoid some pathogenic bacteria (e.g., *C. pneumoniae, H. pylori, A. actinomycetemcomitans*, and *P*. gingivalis) entering into the bloodstream and reaching different tissues, in which they can promote CMD through immune system elicitation, host metabolic and inflammatory response regulation.^118–120^ A correct modulation of the mucus layer thickness is also important for the epithelial barrier function. Butyrate can increase the production of MUC2, a predominant mucin glycoprotein secreted by goblet cells.^121,122^ Finally, SCFAs can promote the antimicrobial peptide secretion by the IEC. For instance, SCFAs promote the RegIIIγ and defensins production by activating mTOR and STAT3, and thus regulating the epithelial barrier functions.^123^

### Energy intake and energy use

SCFAs might present positive effects on body weight control by regulating the energy intake and energy expenditure. Some insights have been obtained into the mechanisms by which SCFAs regulate appetite. A potential mechanism might be the stimulation of secretion of gut-derived satiety hormones, such as peptide YY (PYY) and glucagon-like peptide 1 (GLP-1), by SCFAs binding to the free fatty acid receptor FFAR2 and FFAR3.^124^ Both hormones, which are secreted by intestinal enteroendocrine L-cells,^125^ influence appetite by activating proopiomelanocortin (POMC) neurons in the hypothalamic arcuate nucleus, suppressing neuropeptide Y (NPY), and delaying or inhibiting gastric emptying.^126–128^ Expression of genes encoding PYY is also regulated by receptor-independent pathways. Indeed, the inhibitory activity of HDAC by butyrate leads to an increased PYY expression in human L-cells.^129^ Besides, a study using in vivo^11^C-acetate and PET-CT acetate demonstrated that acetate can cross the blood-brain barrier and is taken up by the hypothalamus, causing an appetite decrease and increase of γ-aminobutyric acid and lactate.^130^ The secretion of leptin, which is often referred to as the “satiety hormone”, might be also stimulated by SCFAs, resulting in a decreased appetite.^131,132^ For instance, human adipocytes incubated with a high concentration of propionate appeared to increase the leptin mRNA expression and leptin secretion.^133^

### Glucose homeostasis and insulin resistance

Several studies have suggested that SCFAs can improve glucose homeostasis in vivo by controlling blood glucose levels and increasing glucose uptake mediated by FFAR2 and FFAR3 activation.^111,112,134^ Although the mechanisms are not completely clear, such effects might happen directly via an AMP-activated protein kinase (AMPK)-dependent co-regulated pathway or indirectly via the PPY and GLP-1 hormones. Indeed, Li *et al*. (2019)^135^ have reported that butanoate can affect glucose metabolism through the up-regulation of AMPK-dependent gene expression. Another study has shown that propionate declines hepatic gluconeogenesis via the same mechanism.^136^ Furthermore, apart from the previously commented functions of PYY and GLP-1, PYY can also contribute to the glucose clearance in adipose tissue and muscle, and GLP-1 can increase insulin secretion and decrease glucagon secretion by the pancreas, regulating blood glucose levels.^137^ At the same time, it seems that SCFAs can exert anti-diabetic effects in the host. Propionate presents benefits on pancreatic β-cell function in vivo, and enhances glucose-stimulated insulin release via FFAR2 activation and increases β-cell mass.^134^ Besides, the binding of SCFAs to the FFAR2 receptor might ameliorate insulin resistance by promoting autophagy of skeletal muscle cells.^138^

### Lipid metabolism

SCFAs can regulate lipolysis and adipogenesis. Acetate and propionate may inhibit endogenous lipolysis, whereas propionate can regulate extracellular lipolysis mediated by an increase of lipoprotein lipase expression, both cases resulting in a decrease of the circulating lipid plasma levels and body weight.^132,139^ As well, SCFAs might play an important role in adipogenic differentiation. Indeed, preadipocytes treated with propionate, and acetate promoted adipocyte differentiation, via an overexpression of FFAR2 and PPARγ.^140,141^ Finally, acetate, propionate, and butyrate seem to enhance hepatic uptake of cholesterol from the blood, decreasing plasma cholesterol in model animal studies.^142,143^ Besides, propionate is a potent inhibitor of cholesterol synthesis.^144^

### Immune function and anti-inflammatory response

SCFAs play a role in the immune system regulation. Of note, it has been shown that butyrate can inhibit HDAC and the activation of nuclear factor kappa β (NF-κB) in macrophages.^145,146^ Both, HDAC and NF-κB, contribute to the immune and inflammatory response.^147^ SCFAs are also involved in anti-inflammatory response by up-regulating anti-inflammatory cytokines and downregulating pro-inflammatory ones. For example, SCFAs binding to FFAR2 and GPR109A in IEC stimulates K+ efflux and hyperpolarization, leading to the inflammasome-activating protein NLRP3 activation, and thus, inducing the IL-18 release, which helps in the maintenance of integrity, repair, and intestinal homeostasis.^148,149^ Increased protein acetylation and production of TGFβ1 in IEC by butyrate lead to a decrease of IL-8 production in IEC^150^ and a promotion of anti-inflammatory regulatory T cells (Treg),^151^ respectively. In human mature dendritic cells, butyrate and propionate appear to reduce the release of pro-inflammatory chemokines, such as CXCL11, CXCL10, CXCL9, CCL5, CCL4, and CCL3, just as inhibiting the expression of LPS-induced cytokines, including IL-6 and IL-12p40.^152^ Apart from the cytokine production regulation, the luminal pH reduction by SCFA inhibits the growth of pathogenic bacteria.^153^ Lastly, SCFAs, specifically butyrate, can contribute to host defense by inducing the antimicrobial protein cathelicidin IL-37^154,155^ and increase the levels of T regulatory cells in the gut.^156^

Taking all this together, we can deduce that SCFAs can exert benefits in CMD, which are characterized by a deregulation of the blood pressure, glucose, and lipid metabolism, inflammation response, and/or gut barrier integrity. Indeed, several studies have demonstrated the benefits exerted by SCFAs in CMD. **Table 1** shows some of these studies.

**Table 1.** Summary of studies reporting beneficial effects of SCFAs in cardio-metabolic health through different traits

Phenotype	Trait	Study	Study design	Mechanism/ SCFA-producing bacteria	Main results
Atherosclerosis	Blood pressure	Li (2018) ^157^	HUVEC pre-incubated alone or with FFAR2 and FFAR3 antagonists and then exposed to TNFα or LPS	SCFA-mediated activation of FFAR2 and FFAR3, and HDAC inhibition	- Acetate: ↓ IL-6 and IL-8 - Propionate and butyrate: ↓ IL-8, VCAM-1 and PBMC adhesion to endothelial monolayer
Cardiovascular diseases	Inflammation	Bartolomaeus (2019) ^158^	Wild-type NMRI or apolipoprotein E knockout–deficient mice receiving propionate or control	T-cell dependent	Both models: ↓ systemic inflammation, cardiac hypertrophy, fibrosis, vascular dysfunction and hypertension
Obesity	Gut barrier integrity and energy usage	Kang (2017) ^159^	Mice fed a high-fat diet supplemented with capsaicin	↑*Ruminococcaceae* and *Lachnospiraceae*	↓ metabolic endotoxemia and body weight gain
	Gut barrier integrity and inflammation	Cani (2007) ^160^	High-fat-diet-fed mice using oligofructose or control	↑*Bifidobacterium* spp.	- ↓ endotoxemia and proinflammatory cytokines (plasma and adipose tissue) - Improvement: glucose tolerance, glucose-induced insulin secretion
	Inflammatory response and lipid metabolism	Schneeberger (2015) ^155^	Diet-induced obesity male C57BL/6 J mice	*Akkermansia muciniphila*	↓ inflammation, metabolic disorders, altered adipose tissue metabolism
	Metabolic homeostasis	Dao (2016) ^161^	49 overweight and obese adults with calorie restriction	*Akkermansia muciniphila*	Improvement: insulin sensitivity markers and metabolic status
	Metabolic homeostasis	Kimura (2013) ^162^	GPR43-deficient and GPR43-overexpressing mice	SCFA-mediated activation of GPR43	↓ fat accumulation (adipose tissue) ↑ lipid and glucose metabolism (other tissues)
	Energy intake	Lin (2012) ^163^	C57BL/6 N male mice receiving SCFAs	FFAR3-independent	Butyrate and propionate: ↓food intake and body weight
	Insulin resistance	Gao (2009) ^164^	Dietary-obese C57BL/6 J mice fed with butyrate	Energy expenditure promotion and mitochondria function induction	↑ thermogenesis and fatty acid oxidation – Prevention of insulin resistance and obesity development
T2D	Metabolic homeostasis	Sakakibara (2006) ^165^	Diabetic KK-A mice fed with acetic acid and control	AMPK in the liver	↑ gluconeogenesis and lipogenesis genes
	Metabolic homeostasis and blood pressure	Roshanravan (2017) ^166^	60 patients with T2D receiving (A) sodium butyrate capsules, (B) inulin supplement powder, (C) inulin and sodium butyrate and (D) placebo	GLP-1	- Group A,B,C: ↓ reduced diastolic blood pressure - Group C: ↓ fasting blood sugar and waist to hip ratio

AMPK, AMP-activated protein kinase; CMH, cardio-metabolic health; FFAR, free fatty acid receptor; GLP-1, glucagon-like peptide-1; GPR43, G-protein coupled receptor 43; HUVEC, human umbilical vein endothelial cells; IL, interleukins; HDAC, histone deacetylase; SCFA, short-chain fatty acid; OLETF, Otsuka Long-Evans Tokushima Fatty; PBMC, peripheral blood mononuclear cell; PYY, peptide YY; T2D, type-2 diabetes; VCAM-1, vascular cell adhesion molecule-1.

**Table 1**. Summary of studies reporting beneficial effects of SCFAs in cardio-metabolic health through different traits.

## Interplay between diet, gut microbial SCFAs, and CMD

Diets rich in fiber and omega-3 exert beneficial effects in CMD^167,168^ lowering the risk of CVD by about 30%, for example, in the Mediterranean diet.^168^ The risk decrease is due to the capability of these diets to reduce risk factors associated with CMH, such as blood pressure, cholesterol, body weight, and systematic inflammation.^65,108,169,170^ Moreover, short- and long-term diets can induce shifts in gut microbiota activity and composition, and thus, in the SCFA synthesis profile.^171–173^ The interplay between diet, gut bacteria-derived metabolites, specifically SCFAs, and CMD has only been reported recently. **Table 2** depicts some studies in which this interplay is shown. As some authors have suggested,^65,108,186^ dietary interventions are a low-risk and cheap strategy to modulate the gut microbiota composition, particularly toward an augmented SCFA production providing benefits to CMH.

**Table 2.** Studies reporting the modulation by diets of SCFA-producing bacteria, SCFA levels, and risk factors associated with cardio-metabolic health

Diet type	Study	Subjects	Dietary design	Modulation of SCFA-producing bacteria and/or SCFA levels	Risk factors associated with CMD
Fiber	Djekic (2020) ^169^	27 ischemic heart disease patients	4 weeks. Vegetarian diet (fiber-rich diet) vs meat diet	↑ *Ruminococcaceae*, *Lachnospiraceae* and *Akkermansiaceae*	↓ body weight, total, LDL and oxidized LDL cholesterol
	Medina-Vera (2019) ^65^	81 T2D patients	3-months. Fiber-rich diet	↑ *F. prausnitzii* and *A. muciniphila*	↓ glucose, total and LDL cholesterol, FFA, HbA1c
	Kasahara (2018) ^174^	Germ-free apolipoprotein E-deficient mice colonized with synthetic microbial communities (different capacities to generate butyrate)	16 weeks. Dietary plant polysaccharides	*Roseburia*	↑ fatty acid utilization ↓systematic inflammation → amelioration of atherosclerosis development
	Zhao (2018) ^175^	33 T2D patients	84 days. Standard diet for T2D patients (C) vs fiber-rich diet	↑ acetate- butyrate- producing bacteria strains	↓ HbA1c via ↑ GLP-1 production
	Marques (2016) ^108^	Sham and mineralocorticoid excess–treated mice	3 weeks. Control diet vs high-fiber diet and acetate supplementation	↑ acetate-producing bacteria (indep. mineralocorticoid excess)	↓ gut dysbiosis, SBP, DPB, cardiac fibrosis, left ventricular hypertrophy, Egr1, renin-angiotensin (kidney), MAPK (heart)
	Weitkunat (2015) ^176^	Obesity induced by high-fat diet in gnotobiotic C3H/HeOuJ mice colonized with a simplified human microbiota	6 weeks. High-fat diet supplemented either with 10% cellulose (C) or inulin	↑ total SCFAs in cecum and portal vein plasma ↓ cecal acetate:propionate ratio	↓ hepatic expression of genes involved in lipogenesis and fatty acid elongation/desaturation
	Kim (2013) ^177^	6 obese subjects with T2D and/or hypertension	4 weeks. Strict vegetarian diet	↑ *B. fragilis, Clostridium* spp. (clusters XIVa and IV)	↓ body weight, total, LDL cholesterol, triglycerides, HbA1c, fecal Lcn-2 ↑ fasting and postprandial glucose
	Filippo (2010) ^178^	Comparison between children from a rural African village and European children	Rural African children: ↑ fiber intake than European children	↑ Bacteroidetes ↑SCFAs	-
	Gutiérrez-Díaz (2016) ^179^	31 healthy individuals	6 months. Mediterranean diet vs lower fiber diet	↑ propionate and butyrate concentrations in feces ↑ *Bifidobacterium, Faecalibacterium*	-
omega-3	Robertson (2017) ^170^	C57BL/6 female mice and male offspring	Gestation (female), 12 weeks (offspring). n-3 PUFA deficient (-n-3) vs supplement (+n-3) vs control	- n-3: ↓*Clostridiaceae*	+ n-3 PUFA: ↑ cecal metabolites involved in energy metabolism
	Watson (2018) ^66^	22 healthy middle-aged subjects	2 months. Treatment with omega-3 PUFA capsules or drinks	Both formulations: ↑ *Lachnospira, Roseburia, Lactobacillus* and *Bifidobacterium*	-
	Menni (2017) ^180^	876 middle aged and elderly female twins	Estimated food intake of omega-3 PUFA: Food frequency questionnaires	↑DHA serum levels: ↑ *Lachnospiraceae*, *Ruminococcaceae* and Bacteroidetes	-
	Balfegó (2016) ^181^	35 drug-naïve patients with T2D	6 months. Standard diet for T2D patients (C) vs same + 100 g sardines (5 days a week)	↑ Bacteroides and Prevotella	-
	Noriega (2016) ^182^	45-year-old male	2 weeks. Omega-3 rich diet (600 mg/day)	↑ *Eubacterium, Roseburia, Anaerostipes, Coprococcus, Subdoligranulum* and *Pseudobutyrivibrio*	-
Dairy products fermented with beneficial bacteria	Veiga (2010) ^183^	T-bet−/−Rag2−/− mice	*Bifidobacterium animalis subsp. Lactis* DN-173 010 strain fermented milk	↑ cecal SCFA ↑ butyrate-producing bacteria	↓ inflammation
	Veiga (2014) ^184^	32 women (aged 20–69 years)	4 weeks. Consumption of (125 g/serving) twice a day of either the *Bifidobacterium animalis* fermented product (n = 17) or acidified milk product (C) (n = 15)	↑ colonic SCFA ↑ butyrate-producing bacteria (MGS126 and MGS203)	-
	Zhang (2013) ^185^	50 healthy adult volunteers	4 weeks. 12% (wt/vol) skimmed milk supplemented with 10(10) CFU *Lactobacillus paracasei* subsp. *Paracasei LC01* (n = 25) each day vs skimmed milk (C) (n = 25)	↑ *Lactobacillus, Bifidobacterium* and *Roseburia* *↑* acetic acid and butyrate acid levels	-

C, control; CMH, cardio-metabolic health; DBP, diastolic blood pressure; Erg-1, early growth response protein 1; FFA, free fatty acids; GLP-1, glucagon-like peptide-1; HbA1c, hemoglobin A1c; MAPK, mitogen-activated protein kinase; Lcn-2, lipocalin-2; LDL, low density lipoprotein; PUFA, poly-unsaturated fatty acids; SBP, systolic blood pressure; SCFA, short-chain fatty acid; T2D, type-2 diabetes. NOTE: When the type of diet is not specified in the last two columns, then the observed effects are associated with the diets under study (1st column).

The modulation of SCFA-producing bacteria and/or SCFA levels by three different diets, namely, fiber-rich diets, omega-3 rich diets, and dairy products fermented/supplemented with beneficial bacteria, just as the exerted benefits in CMH are discussed below.

### Fiber-rich diets

Inulin, resistant starch, fructo-oligosaccharides, and polysaccharides, among others, present the food label of fiber in the US.^187^ It has been suggested that fiber-rich diets, such as the Mediterranean and vegetarian diets, can protect against the development of CMD, including CVD, obesity, and T2D, by modulating the gut microbiota.^188^

Many studies have shown that SCFA concentrations and/or SCFA producing-bacteria can be enhanced by fiber-rich diets. Indeed, a transversal study with 31 healthy individuals showed that the individuals following a 6-month Mediterranean diet presented higher propionate and butyrate concentration in feces, and higher levels of *Bifidobacterium* and *Faecalibacterium* compared to those having a lower fiber intake.^179^ Another study comparing the fecal microbiota between children from a rural African village with European children observed that African children, who have fiber-rich diets, presented significantly more SCFAs and bacteria belonging to the Bacteroidetes phylum.^178^ More studies in which fiber-rich diets positively influence SCFA production can be found in the review conducted by Dreher (2017).^189^

Other human and murine studies have reported that dietary fiber can improve the risk factors associated with CMD by modulating the SCFA-producing gut bacteria. For instance, after following a 3-month fiber-rich diet, patients with T2D showed an increase of the SCFA producing-bacteria *F. prausnitzii* and *A. muciniphila*, as well as a decrease in glucose, total and LDL cholesterol, free fatty acids and hemoglobin A1c (HbA1c),^65^ suggesting that a long-term adherence to a high-fiber diet might improve dyslipidemia, glycemic control, and inflammation by increasing SCFA-producing bacteria. In another study, a vegetarian diet assigned to patients with ischemic heart disease caused an increase of the families *Ruminococcaceae*, *Lachnospiraceae*, and *Akkermansiaceae*, and a decrease of LDL and total cholesterol and body weight compared to the patients following a meat diet.^169^ Therefore, it was suggested that the improvement of these cardiometabolic risk factors might be caused by the capability of these bacterial families to produce SCFAs. Finally, a murine model study showed that *Roseburia* (butyrate-producing bacterial genus) can interact with dietary plant polysaccharides, promoting fatty acid utilization and reducing systematic inflammation, which turns in the amelioration of atherosclerosis development.^174^

### Omega-3 rich diets

Omega-3 rich diets have been also associated with an increase of SCFA-producing bacteria. Indeed, in a randomized trial, 2-month of treatment with omega-3 polyunsaturated fatty acids (PUFA) capsules or drinks was given to 22 healthy middle-aged subjects to analyze the effect of omega-3 PUFA on the human gut microbiota.^66^ An increased abundance of SCFA-producing bacteria belonging to the *Lachnospira, Roseburia, Lactobacillus*, and *Bifidobacterium* genus was reported in the subjects taken one or both formulations (capsules and drinks). Noriega et colleagues (2016)^182^ showed a significant increase of the butyrate-producing bacteria *Eubacterium, Roseburia, Anaerostipes, Coprococcus, Subdoligranulum*, and *Pseudobutyrivibrio* after 2 weeks of an omega-3 rich diet.

Other studies have also reported this association and suggested the potential beneficial role of this type of diet in CMD. For instance, a recent human study showed a significant increase in *Coprococcus* spp. *and Bacteroides* spp., and in isobutyrate and isovalerate levels after a daily supplementation of 500 mg of omega-3 fatty acid for 6 weeks.^190^ Likewise, *Coprococcus* was positively associated with the branched-chain fatty acid isobutyric acid and negatively associated with the triglyceride-rich lipoproteins VLDL and VLDL-TG, suggesting that dietary omega-3 influences the gut microbiota composition and its benefits in CVD might be mediated by gut fermentation products.^190^ In another study, three different diets were fed to female mice, namely control, n-3 PUFA supplemented and n-3 PUFA deficient, during gestation, and then, the male offspring continued with the same diet for 3 months.^170^ Deficiency of n3-PUFA in diet reduced the levels of acetate and butyrate in feces, and the Clostridiaceae family, which can produce SCFAs, was not detected in this group. Likewise, the metabolic results revealed an increase of cecal metabolites involved in energy metabolism in n-3 PUFA supplemented mice. Thus, it was suggested that the observed impairment of SCFA production might disrupt the metabolism homeostasis and thus, having an impact on metabolic diseases. In a study conducted by Balfegó and coworkers,^181^ gut bacterial species were determined in drug-naïve patients with T2D before and after following either a standard diet (control) or the same diet but enriched with sardines for 6 months. Individuals following the latter diet presented an increase of *Bacteroides* and *Prevotella* (acetate-producing bacteria) in comparison with the baseline. It was indicated that this diet might present benefits for cardiovascular risk.

### Dairy products fermented or supplemented with beneficial bacteria

A few studies have reported the capability of dairy products fermented or supplemented with beneficial bacteria to increase SCFA-producing bacteria. *Bifidobacterium animalis* subsp. *lactis* fermented milk product resulted in an increase of butyrate-producing bacteria and cecal SCFA in a mouse model, and an inflammation reduction.^183^ This statement was further validated in humans, where the same fermented milk product potentiated colonic SCFA production and increased two previously uncharacterized butyrate producers, namely MGS126 and MGS203.^184^ Another study reported that skimmed milk supplemented with *Lactobacillus paracasei* subsp. *Paracasei* caused in healthy young adults a significant increase in *Lactobacillus, Bifidobacterium*, and *Roseburia* and in the acetic acid and butyrate acid levels, compared with the control (only skimmed milk).^185^

However, more research is required in the CMD field as no study has focused on investigating the effect of these dairy products in these diseases yet.

**Table 2**. Studies reporting the modulation by diets of SCFA-producing bacteria, SCFA levels, and risk factors associated with cardio-metabolic health.

## Conclusions and future directions

The composition, diversity, and activity of the gut microbiota can be easily modified by dietary patterns or specific nutritional components. Indeed, several studies have demonstrated that certain diet types can increase the abundance of SCFA-producing bacteria, leading to an augmented SCFA level. Likewise, SCFAs have shown promising results in the modulation of CMD, as they can improve different risk factors associated to these diseases, such as dyslipidemia, cholesterol, insulin resistance, hyperglycemia, and inflammation, thanks mainly to their capability to inhibit HDAC and activate the different FFAR receptors. However, long-term human intervention studies analyzing the effect of different diets in the SCFA production and the benefits exerted in patients suffering from CMD are clearly needed. These types of studies would contribute to deeply understand the interplay between diet, SCFAs, and CMD, enabling us to design dietary strategies that prevent/improve the CMD development by enhancing the SCFA production. Thus, the major public health challenge that CMD represents might be addressed with these low-risk and cheap strategies. Additionally, a limitation of some human studies is the lack of consistency in their results due to the variability in the baseline microbiota of the participants. Therefore, future work will focus on how to create personalized dietary strategies based on the microbiota of each individual. As well it would be interesting to determine the synergic effect of different diets (e.g., a diet rich in omega-3 and fiber) in the gut microbiota and CMH.

## Abbreviations

AMPK, AMP-activated protein kinase; cardio-metabolic diseases; cAMP, cyclic adenosine monophosphate; CMH, cardio-metabolic health; CVD, cardiovascular diseases; FFAR, free fatty acid receptor; GLP-1, glucagon-like peptide 1; GPR, G-protein coupled receptor; HDAC, histone deacetylase; IEC, intestinal epithelial cells; LPS, lipopolysaccharides; NF-κB, nuclear factor kappa β; PUFA, polyunsaturated fatty acids; PYY, peptide YY; PPARγ, peroxisome proliferator-activated receptor γ; SCFA, short-chain fatty acid; TMA, trimethylamine; TMAO, trimethylamine‑N‑oxide; T2D, type-2 diabetes; Olfr72, olfactory receptor-78.
